# Fine Piercing of Amorphous Electrical Steel Sheet Stack by Micro-/Nano-Textured Punch

**DOI:** 10.3390/ma15051682

**Published:** 2022-02-23

**Authors:** Yukiya Komori, Yohei Suzuki, Kohta Abe, Tatsuhiko Aizawa, Tomomi Shiratori

**Affiliations:** 1 Graduate School of Engineering, University of Toyama, Gofuku, Toyama 930-8555, Japan; m2171214@ems.u-toyama.ac.jp; 2 Komatsu-Seiki Kosakusho Co., LTD., Suwa-City, Nagano 392-0012, Japan; y-suzuki@komatsuseiki.co.jp (Y.S.); k-abe@komatsuseiki.co.jp (K.A.); 3 Surface Engineering Design Laboratory, SIT, 3-15-10 Minami-Rokugo, Ota-City, Tokyo 144-0045, Japan; taizawa@sic.shibaura-it.ac.jp; 4 Faculty of Engineering, University of Toyama, Toyama 930-8555, Japan

**Keywords:** nano-structured punch, fine-piercing, electrical amorphous steel sheets, iron loss

## Abstract

The periodic nanotexture was superposed to the micro-textured grooves on the side surface of the punch. These grooves with nanotextures were shaped to have parallel and vertical orientations to the punch stroke direction, respectively. A stack of five amorphous electrical steel sheets was punched out with these micro-/nano-textured punches. The process affected zone at the vicinity of the punched hole was analyzed by SEM (Scanning Electron Microscopy) and a three-dimensional profilometer. The punch surfaces were also observed by SEM to describe the debris particle adhesion on them. The dimensional change in each layer of the stack before and after perforation was measured to describe the punching behavior with the comparison to the punch diameter.

## 1. Introduction

A high-capacity motor core with low iron loss has been strictly demanded by automotive companies with an urgent shift of production to electric vehicles [1]. As their essential parts, the motor-core is made from a stack of pierced electrical steel sheets. In particular, an amorphous electrical steel sheet is highlighted and selected recently to improve magnetic performance and to reduce the iron losses by 1/10 in comparison to polycrystalline electrical steels [2,3]. These amorphous work materials for piercing have no ductility but are high strength enough to induce many defects in them in piercing even by using a cylindrical punch. In particular, microscopic defects and damages are easily left as an affected zone at the vicinity of the pierced holes even without visible flaws in macroscopic observation. They have a risk to deteriorate the quality of magnetic properties and increasing the iron loss in total [4]. In addition, as had been studied in [5,6,7], this affected zone increases by piercing, and the shearing tool life was significantly reduced to lower the cost-competitiveness. Furthermore, this affected zone was broadened when piercing the stack of sheets [8]. For example, when punching out a stack of five layered amorphous electrical steel sheets by using the normally ground WC (Co) tools, many cracks were induced in the circumferential direction in each constituent sheet [9]. Non-traditional tooling is required to reduce these affected zones and to allow fine piercing of an amorphous electrical steel sheet stack.

As the first new tooling approach, ion-milling was selected to sharpen the WC (Co) punch and die edges to enhance the stress concentration in piercing and to reduce tool wear and the affected zone widths [10,11]. A laser-trimming method was invented as the second approach to reduce the top surface roughness of piercing punch and to form the nano-textures on side surfaces [12]. The affected zone width significantly decreased in piercing the AISI316L sheet with a thickness of 200 µm. The piercing tool life was pro-longed by plasma-nitriding the SKD11 punches [13]. Recently, a diamond coated WC (Co) punch with its nano-textured side surface was successfully utilized in piercing the stack of five amorphous electrical steel sheets to reduce the affected zone widths [14].

In the present study, two types of nitrided SKD11 punches are utilized to investigate the effect of micro-/nano-texturing on the piercing process. The mechanically ground WC (Co) punch is employed as a reference. The stack of five amorphous electrical steel sheets is prepared for the work. The fine piercing experimental setup is developed to analyze the shearing effect on the induced defects by piercing. SEM and a three-dimensional profilometer are utilized to measure the affected zone width and work sheet surface profiles.

## 2. Materials and Methods

The fine piercing procedure is explained with some notices on the shearing and tooling conditions. The pierced electrical amorphous steel sheets are analyzed to describe the effect of micro-/nano-textures on the punch side surface on the quality of pierced holes.

### 2.1. Stamping Procedure

**Figure 1:**
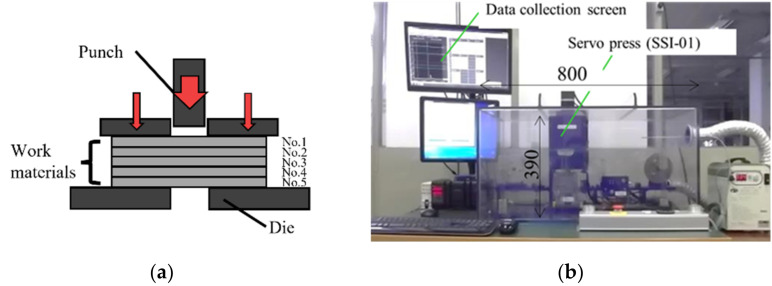
A small-scaled screw servo-stamping system. (**a**) A schematic view on the piercing procedure of amorphous electrical steel sheet stack, and (**b**) outlook of system.

An electrical amorphous steel sheet with a thickness of 25 μm was used as a workpiece. Five pieces were laminated into a stack; this stack was employed as a work for piercing experiments as shown in Figure 1a. A small-scaled screw servo-stamping system with the loading capacity of 10 kN (SSI01 system, Microfabrication Laboratory, LLC.; Tokyo, Japan), was used for the present piercing experiment in dry without lubrication as shown in Figure 1b. The punching speed was 5.0 mm/s. The piercing load was measured by the load cell (LMA-S-50N, Kyowa Electric Co., Ltd.; Tokyo, Japan) which was embedded into the lower die set. The stroke was measured by the laser displacement meter (LK-G30, KEYENCE Co., Ltd.; Tokyo, Japan). The whole measured data were transferred to PC through the interface (NR-600, KEYENCE Co., Ltd.; Tokyo, Japan). The holding load was fixed by 1.3 kN.

### 2.2. Tooling Conditions

**Figure 2:**
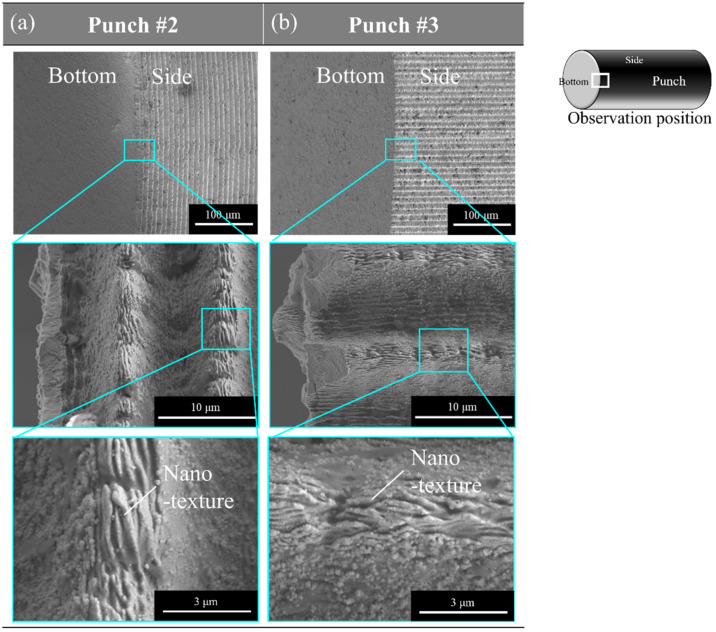
SEM images of the microstructure of the punch top and side surfaces across the punch edge for punch #2 and #3.

Three punch–die pairs were prepared for tooling as listed in Table 1. The normal punch (punch #1) and die were made from WC (Co) with the ground finish for reference. Two other punches (punches #2 and #3) were made of plasma-nitrided SKD11 with two types of nano-textures on their side surfaces. Figure 2 shows the SEM images on their punch side surfaces with micro-grooves and nano-textures. They were wrought by ultra-short pulse laser machining. The circumferential micro-grooves with their pitch of 10 µm and their depth of 8 µm were laser-textured into punch #2. Nanotextures were formed by LIPSS (Laser Induced Periodic Surface Structuring) during this laser microtexturing. They were also aligned in the circumferential micro-texture with the LIPSS-period of 300 nm. Punch #3 had micro-grooves, aligned along the punch stroke direction in the pitch of 10 µm and their depth of 8 µm. Nanotextures were also aligned in the punch stroke direction on the micro-textured side surface with the LIPSS-period of 300 nm. The maximum diameter was 1.981 mm for punch #2, and 1.985 mm for punch #3, respectively. An argon ion milling system (IM-4000, Hitachi Co., Ltd.; Tokyo, Japan) was utilized to polish and shave the inner cutting edge of two SKD11 dies, respectively, for punches #2 and #3. This edge width was sharpened to be less than 1 µm. The die hole diameter for piercing was 2.003 mm against punch #2, and 2.004 mm against punch #3, respectively. Either side clearance between the punch and the die was 11.0 µm for punch #2 and 9.5 µm for punch #3, respectively.

### 2.3. Work Materials

An amorphous electrical steel sheet with a thickness of 0.025 mm was used for this experiment. A test-piece with the half size of JIS No. 13B standard piece was prepared for uniaxial tensile testing. The maximum tensile strength was 2.2 GPa at an elongation of 1.09% [14].

### 2.4. Characterization of the Pierced Works

The pierced work sheet surface was analyzed by the SEM (JSM-6060KU; JEOL Co., Ltd., Tokyo, Japan). In addition, the three-dimensional surface profile was also analyzed by using the non-contact three-dimensional profiling system (NT91001, Bruker AEX Co., Ltd.; Tokyo, Japan). In the following, the right-side surface of the pierced hole in each amorphous sheet was selected for measurement.

## 3. Experimental Results

### SEM Analysis on the Affected Zones

**Figure 3:**
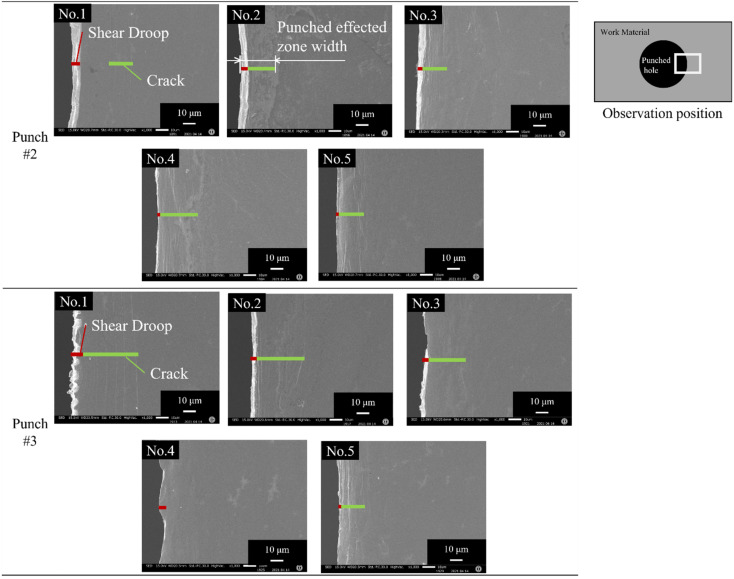
SEM images on the right-side surface of the first, the third and fifth perforated amorphous electrical steel sheets in the pierced stack.

Each amorphous electrical steel sheet of the pierced stack was analyzed by SEM to measure its affected zones by piercing. Figure 3 shows the SEM images on the right-side surface of the first to fifth perforated sheets in the pierced stack. When using punch #2, two types of defects were induced to the first sheet in the pierced stack, e.g., a shear droop at the edge of the pierced hole, and a circumferential crack along the hole. The cracked zone width varies from one sheet to the other in Figure 3. The shear droop was commonly detected in the whole sheets and its width was smaller than 10 µm. The total affected zone width, including the shear droop and cracked zone, was 25 µm for each pierced sheet. When using punch #3, a wavy distortion was also noticed near the hole of the first sheet in addition to these two defects, while no wavy distortions were seen in the third sheet. On the other hand, these three defects were seen in every sheet of the stack when using punch #1. Later, we explain how the circumferential cracks and the wavy distortion are induced by the piercing process when using the punches with different edge conditions.

**Figure 4:**
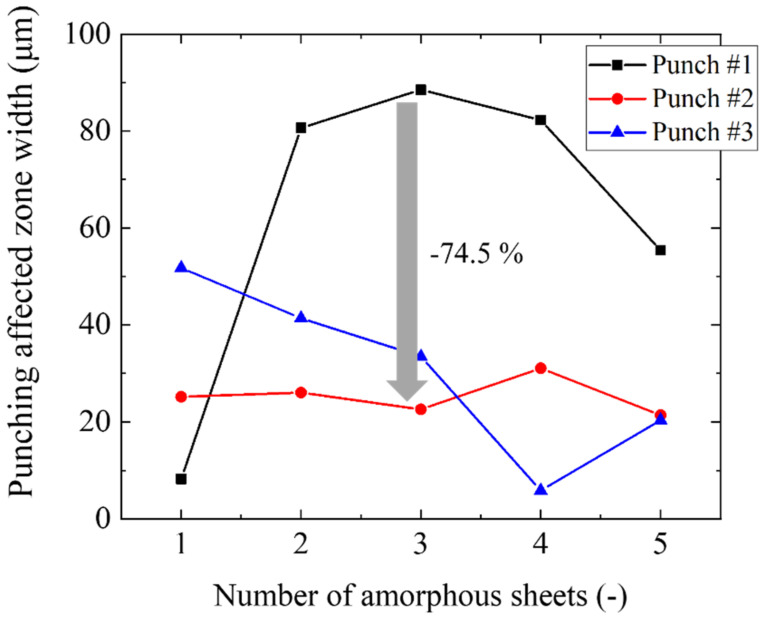
Variation of the punching affected zone width in each pieced amorphous electrical steel sheet in the stack when using punch #1, #2 and #3.

Figure 4 compares the measured punching affected zone widths of five pierced sheets when using punch #1, #2 and #3, respectively. The zone width of the fifth sheet is strongly affected by the die edge. The first to fourth pierced sheets are representative of the affected zone width by punching. When using punch #1, a small width of 9 μm for the first sheet jumped up in the second to fourth sheets. In the case of punch #2, the affected zone width was nearly constant by 25 μm from the first to the fifth sheet. When using punch #3, the highest affected zone width decreased monotonously from the first to the fourth sheet, e.g., it reached down to 7 μm at the fourth sheet. This significant difference in the affected zone width among the three punches, reveals that the micro-/nano-textures on the side surface of the punch have a significant influence on the piercing behavior. Let us evaluate this difference among the three punches in the third work piece. When using punches #2 and #3, the affected zone widths are nearly the same as 20 μm. While it became 90 μm, more than the original amorphous sheet thickness by three times when using punch #1. The affected zone width was saved by 74.5% only by changing the normal ground WC (Co) punch to the nitrided SKD11 #2 and #3 punches with micro-/nano-textures.

**Figure 5:**
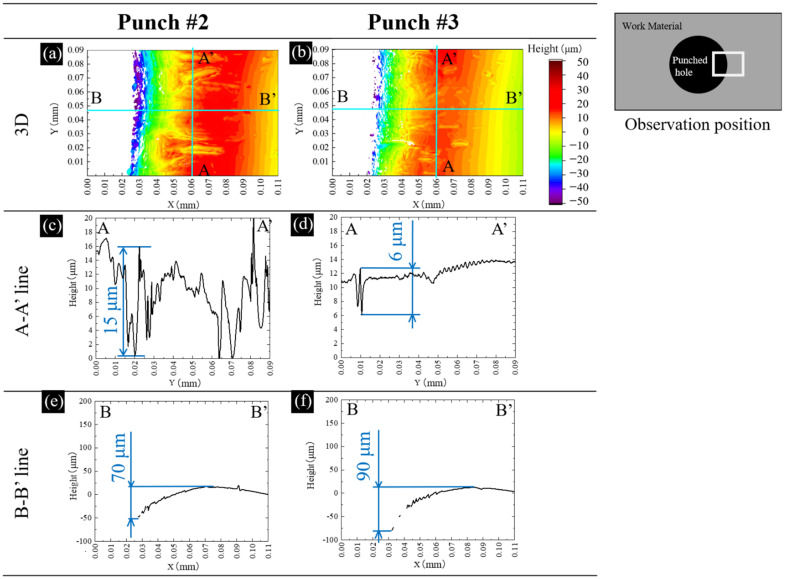
Comparison of three-dimensional profiles for each pierced sheet at the vicinity of the pierced hole in the right side surface of the third sheet between two punches.

Let us measure the three-dimensional profile at the vicinity of the pierced hole on the right-side surface of the third sheet. As depicted in Figure 5a,b, the amorphous sheet is sheared and compressed to deform the flat surface to this convex profile. This convex profile by piercing punches #2 and #3, differs from each other. Two cross-sections were selected to analyze two convex profiles for punch #2 and #3, respectively. The A–A’ cross-section represents the surface profile in the circumferential direction. The B–B’ cross-section describes the height elevation in the lateral direction. When using punch #2, many deep peaks and valleys are noticed in Figure 5c while a few shallow valleys with the maximum depth of 6 μm were only seen in Figure 5d. These pairs of peaks and valleys are caused by wrinkling distortion. In comparison, severe distortions were seen when using punch #1, the damage by the wrinkling distortion is reduced when using punches #2 and #3. Nearly flat A–A’ cross-sectional profile in Figure 5d reveals that the piercing process by punch #3 is free from the wrinkling distortion. The B–B’ cross-sectional profiles in Figure 5e,f include the geometrical change by the shear droop near the hole edge and the discontinuous peaks and valleys. This total height change reaches 60 μm in Figure 5e and 70 μm in Figure 5f, respectively. There is little difference of induced shear droop by piercing with the use of punch #2 and #3.

**Figure 6:**
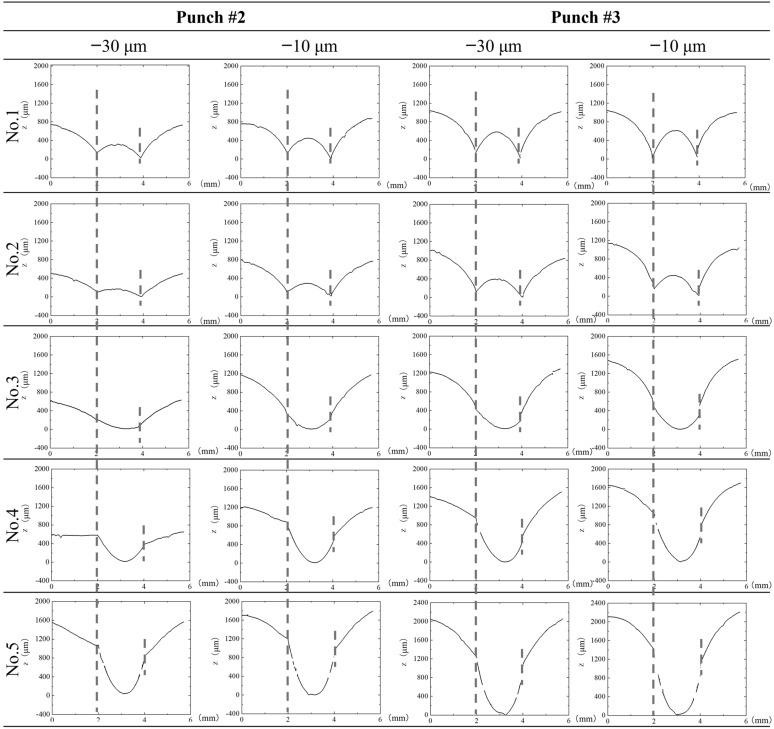
Variation of electrical amorphous steel sheet in the stacked work by controlling the stroke δ = *δ*_final_ − 30 *μ*m and *δ*_final_ − 10 μm by using two micro-/nano-textured punches. δ_final_ denotes the critical stroke when the work is punched out. These deformed sheets are measured by the three-dimensional profilometer from their left side to their right side along the distance of 6 mm.

The transient surface profile of each sheet in the stack was measured by controlling the applied stroke (δ). The punch stroke was applied by *δ* < δ_final_, which is a final stroke to make a complete perforation. In particular, the stroke was terminated to be δ = δ_final_ − 30 μm and δ = δ_final_ − 10 μm to measure each sheet surface profile of stack by using the three-dimensional profilometer. Figure 6 compares the three-dimensional profile of the first to fifth sheets in the stack at a stroke of δ = δ_final_ − 30 μm and δ = δ_final_ − 10 μm, respectively, by using two micro-/nano-textured punches. In common, the first and second sheets both at δ = δ_final_ − 30 μm and δ = δ_final_ − 10 μm, have a W-lettered profile. During the piercing process, the punch edge indents into each sheet and causes the stress concentration to generate the fixed moment at the indented sheet by punch edge. When unloading from the pierced state, this moment is released to spring back the sheet to this W-lettered profile.

In this Figure 6, the broken lines denote the position on each sheet surface to be stressed by the punch edge. This distance (D_H_) between two positions represents the hole diameter to be punched out. Since the first to second sheets are tensiled by indentation of punch, their D_H_-distances are less than the punch diameter (D_p_) due to the elastic spring-back. The third to fifth sheet deformation is also affected by the die edge. Different from the first to second sheets, the third to fifth sheets do not deform in the W-lettered shape but in the U-lettered shape seen in Figure 6. This reveals that shearing takes place earlier in them than the first to second sheets far from the die edge. Let us compare this shearing behavior when using punch #2 and #3. When comparing this U-lettered deformation of the third sheet at *δ*_final_ − 30 μm, this U-letter deformation is accelerated by using punch #3. The deeper U-shape is formed by using punch #3; the shearing process is enhanced when piercing with the use of punch #3.

**Figure 7:**
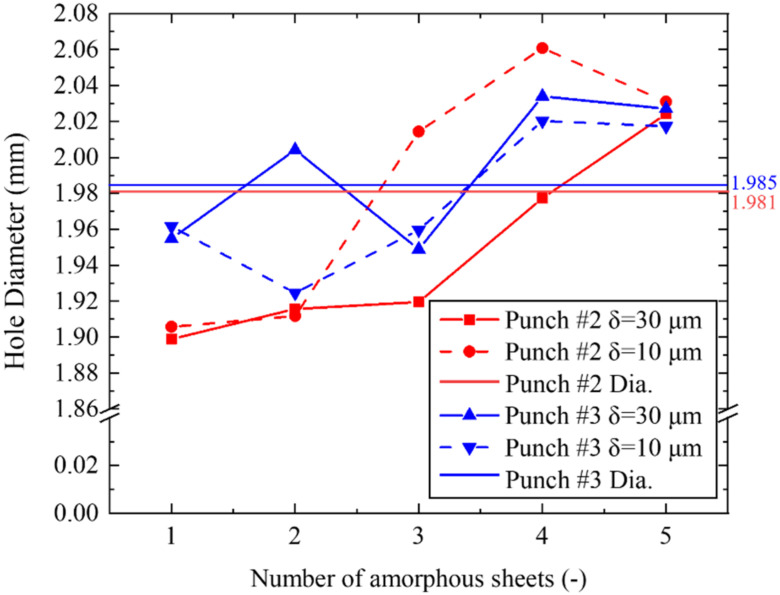
Variation of D_H_-distances in each electrical amorphous steel sheet in the stacked work at the stroke δ = δ_final_ − 30 and δ = δ_final_ − 10 μm before complete perfortaion during the piercing with the use of punch #2 and #3. The puch diamter (D_p_) for punch #2 and #3 are also depicted for reference.

The whole measured D_H_-distances in Figure 6 are summarized in Figure 7 together with the punch diameters (D_p_’s) for punches #2 and #3. When using punch #2, D_H_ < D_p_ for the first to fourth sheets at δ = δ_final_ − 30 μm. D_H_ < D_P_ is only for the first and second sheet, but D_H_ > D_p_ for the third and fourth sheet is at δ = δ_final_ − 10 μm. This implies that the shearing changes local bending within the clearance between the punch and the die edge. In fact, at the fifth sheet in contact to the die edge, D_H_ > D_p_ in every stage, irrespective of the punch indentation. The local bending process governs the sheet deformation near the die edge. When changing punch #2 to #3, the variation of D_H_ for the first to fifth sheets becomes insensitive to the stroke in the punch indentation. D_H_ < D_p_ for the first to third sheets, and D_H_ > D_p_ for the fourth to fifth sheets. In correspondence to the difference in U-shaping for the third to fifth sheets in Figure 6 between two punches, the shearing deformation by the punch indentation governs the whole piercing process when using punch #3. As seen in Figure 6 and Figure 7, the transient state in punching out the stack work before perforation, reveals that the micro-grooves with nanotextures on the punch side surface play an essential role to control the shearing behavior in the piercing process. The shearing process is more enhanced when using punch #3. This proves that the nanotextured punch side surface in parallel to the piercing direction might well be suitable for the fine piercing process.

**Figure 8:**
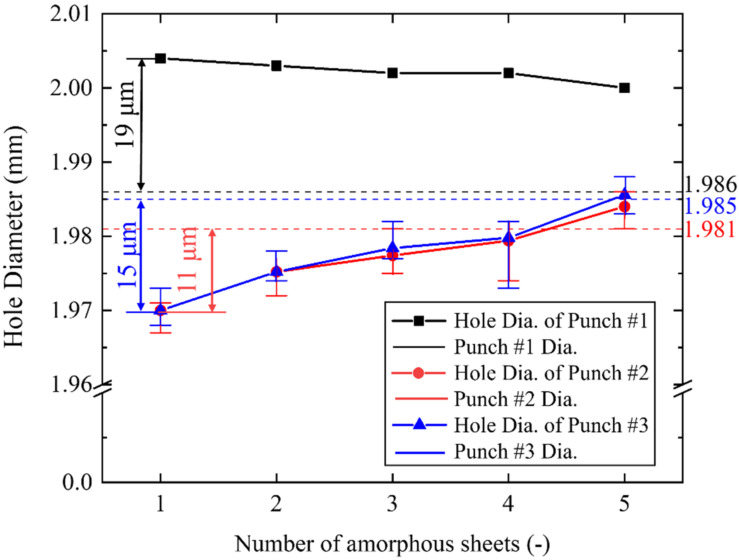
Relationship between the hole diameter (D_N_ for 1 < N < 5) of five sheets and the original punch diamter (Dp) for three punches.

The product quality of the pierced stack work is also determined by the perforated hole diameter. Let us measure each hole diameter in the pierced sheet after the punching test. The pierced hole diameter by using punch #1 was also measured as a reference. The pierced hole diameters by punch #2 and #3 were measured for five samples to deduce their average and deviation. The measured hole diameters (D_N_ for 1 < N < 5) and the punch diameters (D_p_) are compared in Figure 8 among three punches #1 to #3. D_N_ is always larger than Dp irrespective of N when using punch #1. This proves that perforation through the five-layered stack work is propelled by local bending in the clearance, and largely affected zones are induced in each sheet. When using the micro-/nanotextured punch #2 and #3, D_N_ is always smaller than D_p_ irrespective of N. If the piercing process were completely governed by simple shearing, the hole diameter could be equal to D_p_, or, be shortened to be D_N_ < D_p_ by the elastic spring-back. When using the punches with D_p_ ~ 2 mm, this spring-back displacement (Ds) is estimated at maximum by D_s_ = 0.01 × D_p_ = 20 μm. Since D_s_ < 15 μm and D_s_ < 11 μm when using punch #2 and #3, the whole sheet in the stack is sheared by indentation of the punch with less dependency of clearance between the punch and die edges.

**Figure 9:**
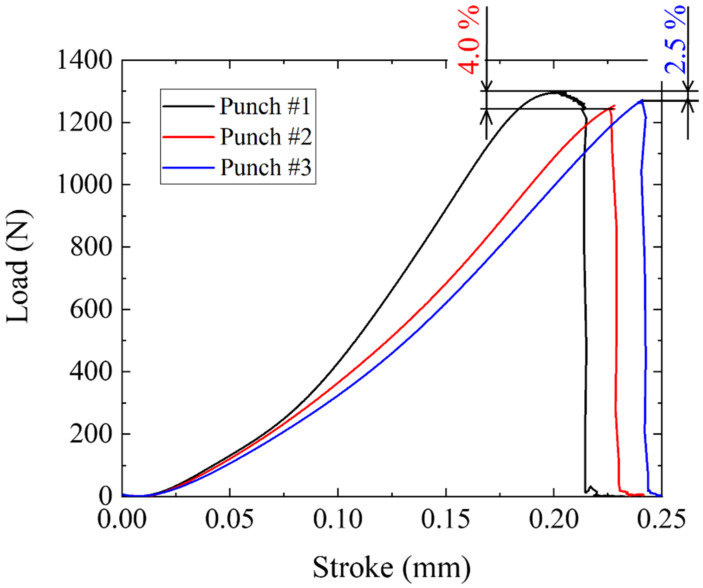
Load–Stroke curve in piercing with the use of three punches.

This difference between D_N_ and Dp reflects on the load–stroke relations in piercing with the use of three punches. As shown in Figure 9, the piercing load increases non-linearly to the maximum load when using punch #1. While this load to stroke curve becomes semi-linear relation up to the maximum load when using punches #2 and #3. The maximum piercing load is reduced by 4.0% when using punch #2, while it decreases by 2.5% in case of the punch #3.

**Figure 10:**
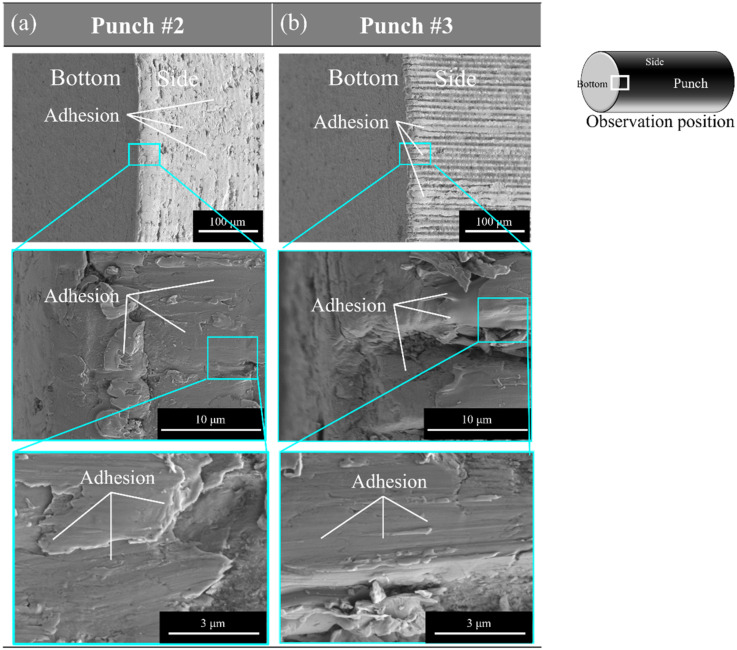
SEM images on the microstructure across the punch edge for punch #2 and #3 after processing. (**a**) SEM images of punch #2, (**b**) SEM images of punch #3.

Figure 10 shows the multi-scale SEM images of the punch edge after punching. When using punch #2, its surface is almost covered by the adhesion of particle debris. On the other hand, from Figure 10b, some adhesion was seen on the top of longitudinal micro-textures. Most of the longitudinal nanotextures are seen on the micro-grooves of punch #3.

## 4. Discussion

In the normal punching process, the multi-axial stresses are applied at the indentation of the dull punch edge into the work. As analyzed in [15], the affected zone by punching widely spreads into the work material. An amorphous electrical steel sheet has high strength and no ductility enough to induce many defects during the metal forming. In the case of piercing with the use of a cylindrical punch, the circumferential cracks and wavy distortions are induced to the blank sheet in addition to the shear droop. In order to reduce this affected zone, the punch edge and punch side-surface topological configurations must be controlled even under relatively large clearance between the punch and die edges. In particular, the punch edge configuration, as well as the punch side surface condition, have a significant influence on the pierced product quality in addition to the punch edge sharpness. When using the mechanically sharpened punch with an inhomogeneous edge profile, the total affected zone width becomes three to four times larger than the amorphous sheet thickness. The shear droop, the wrinkling damage and the cracked zones are left in the pierced sheets. The hole diameter of each pierced sheet is always larger than the punch diameter by 20 μm, nearly equal to the sheet thickness. This large, affected zone width in every pierced sheet, reveals that the piercing process is driven by local bending deformation within the clearance between the punch and die edges. 

When using the micro-/nano-textured punches, the diameter (D_N_; for 1 < N < 5) of pieced sheets is always less than the punch diameter (D_p_). This difference of (D_P_ − D_N_) decreases with N. Although it becomes maximum at N = 1, this (D_p_ − D_N_) for n = 1, is nearly equivalent to the spring-back deformation. This proves that every sheet is simply sheared to have a hole with nearly the same diameter as D_p_, and elastically springs back to be D_N_ < D_p_ after completely punching out. This spring-back deformation increases with increasing the affected zone width when punching the N-layered stack of amorphous steel sheets. The monotonous decrease of (D_p_ − D_N_) with N in the above reveals that the affected zone width is also reduced even by increasing N in the stack. Figure 3 proves that the affected one width is minimized at the fourth and fifth sheets when using punch #3.

The piercing load–stroke relation measures the effect of punch edge configuration and punch side surface condition on the product quality. When using the mechanically ground punch or punch #1 in Figure 8, the load increases linearly in the initial stage for 0 < δ < 0.07 mm with stroke since the pierced blank has no defects. The applied load starts to deviate from the linear relation since a part of the applied power in the piercing is dissipated by the occurrence of damage. Hence, the onset of this deviation in the load–stroke relation is retarded by reducing the damages. When changing the punch from #1 to #2 and #3, this onset is effectively retarded in Figure 9. This also proves that the punch side surface must be controlled to reduce the induced damages by the piercing process.

The mechanically ground punch and die were utilized to have sufficiently narrow clearance between two, in most of the fine piercing operations. This piercing process design [15] stands on the knowledge that the narrow clearance is necessary to prevent the fine piercing from the local bending mode within the clearance and to sustain the shearing mode in the whole piercing operations. When using the edge-sharpened punch by laser-texturing, such as #2 and #3, the fully burnished hole surfaces are attained in the piercing process even under the wider clearances. This proves that the simple shearing mode is sustained even under wider clearance when using the homogeneous punch edge profile and the micro-/nano-textured punch side surface. The stress concentration at the homogeneously sharpened punch edge is needed to improve the product quality and to reduce the affected damages into amorphous steel sheets.

Let us consider how to minimize the affected zone size within the tolerance of products by controlling the nanostructures on the punch side surface. The difference between punch #2 and #3 only lies in the orientation of the micro-grooves and nanotextures at the punch side surfaces. Punch #2 has a circumferential micro-groove; the nanotextures are aligned on the circumferential micro-grooves. Punch #3 has a longitudinal micro-groove along the punching direction; its nanotextures are also aligned on these longitudinal micro-grooves. Figure 6 compares the transient amorphous sheet deformation process till perfect punching-out. The U-letter deformation in the punching direction is enhanced by using punch #3. Considering that the punch #2 edge is circumferentially smooth, the micro-/nano-textured punch #3 edge induces the stress concentration to drive the shear deformation along the punching direction. This suggests that micro-/nano-texturing optimization or optimum design of depth and pitch in texturing has a possibility to further control the shear deformation process. Toward this optimization, each contribution of micro- and nano-textures to the piercing process must be analyzed in further studies.

The micro-/nano-textures on the punches become a dominant factor in the stacked piercing of amorphous electrical steel sheets. Since iron loss in motor cores is induced by residual zones with defects in the electrical steel sheets, this piercing process with the use of micro-/nano-textured punch can directly reduce iron loss by minimizing the affected zone width. In particular, the punch life is also prolonged by using this nano-textured side surface. With further control of depth and orientation, the micro-/nano-textured tool life is also prolonged together with the high qualification of pierced products.

## 5. Conclusions

The micro-/nano-texturing of the piercing punch is a key technology to control the punching characteristics and to improve the quality of pierced products. When using the mechanically ground punch, the affected zone width reaches three times more than the amorphous electrical steel sheet thickness. In addition, the applied power is dissipated in the formation of cracks and distortions to enlarge the applied load–stroke curve. The pieced hole diameter becomes always larger than the punch diameter; those defects are left in the pierced sheets.

This affected zone width is significantly reduced to be much less than the sheet thickness when using the micro-/nano-textured punch. The applied load to stroke relation is reduced to lower the plastic dissipation in the applied power in punching. The pierced hole diameter is always smaller than the punch diameter; this reduction is caused by spring-back during unloading after piercing.

The micro-/nano-texture orientation has an influence on the piercing behavior above. When using the micro-/nano-textured punch along the piercing direction, the debris particles from work are trapped into the nano-grooves to eject them from the piercing process. The plastic dissipation in the applied load to stroke relation is also reduced by controlling the micro-/nano-texture orientation.

Since iron loss in motor cores is induced by residual zones with defects in the electrical steel sheets, this piercing process with the use of micro-/nano-textured punch can directly reduce the iron loss by minimizing of affected zone width. In particular, the affected zone width is uniformly reduced in each amorphous sheet in a stack; high productivity is preserved with high qualification in piercing. Since a normal clearance setup is allowed in this piercing process, the punch and die life is extended without loss of quality in the pierced punch. This high cost-competitiveness is another favorite feature of this micro-/nano-textured tooling.

## Figures and Tables

**Table 1:** Dimensions of three punches.

	Punch #1	Punch #2	Punch #3
Punch Diameter (mm)	1.986	1.981	1.985
Die Inner Diameter (mm)	2.002	2.003	2.004
Clearance (μm)	8.0	11.0	9.5

## Data Availability

MDPI Research Data Policies at https://www.mdpi.com/ethics, accessed on 23 December 2021.
